# Indefinite Pronouns Optimize the Simplicity/Informativeness Trade‐Off

**DOI:** 10.1111/cogs.13142

**Published:** 2022-05-17

**Authors:** Milica Denić, Shane Steinert‐Threlkeld, Jakub Szymanik

**Affiliations:** ^1^ Institute for Logic, Language and Computation University of Amsterdam; ^2^ Department of Linguistics University of Washington at Seattle

**Keywords:** complexity, efficiency, function words, indefinites, informativeness, linguistic universals, semantics, trade‐off

## Abstract

The vocabulary of human languages has been argued to support efficient communication by optimizing the trade‐off between simplicity and informativeness. The argument has been originally based on cross‐linguistic analyses of vocabulary in semantic domains of content words, such as kinship, color, and number terms. The present work applies this analysis to a category of function words: indefinite pronouns (e.g., *someone*, *anyone*, *no one*). We build on previous work to establish the meaning space and featural make‐up for indefinite pronouns, and show that indefinite pronoun systems across languages optimize the simplicity/informativeness trade‐off. This demonstrates that pressures for efficient communication shape both content and function word categories. In doing so, our work aligns with several concurrent studies exploring the simplicity/informativeness trade‐off in functional vocabulary. Importantly, we further argue that the trade‐off may explain some of the universal properties of indefinite pronouns, thus reducing the explanatory load for linguistic theories.

## 1 Introduction

The vocabulary of human languages has been argued to support efficient communication by optimizing the trade‐off between simplicity and informativeness (Kemp & Regier, 2012). In informal terms, the simplicity of a system is a measure of how easy it is to mentally represent that system. The informativeness of a system is a measure of how precisely the system allows us to communicate intended meanings. These two properties of a system trade‐off against each other (Ferrer i Cancho & Solé, 2003; Kemp & Regier, 2012; Rosch, 1978; Zipf, 1949). The reason is that, in general, the fewer expressions a language has, the fewer semantic distinctions it is able to make, but the easier it will be to mentally represent. In other words, simplifying the language often entails sacrificing informativeness, and improving informativeness often requires added complexity. That the category systems of natural languages are (near‐)optimal solutions to trading off these two measures has been originally argued based on cross‐linguistic data and generalizations coming from the semantic domains of content words: kinship terms, color terms, container terms, and number terms (Kemp & Regier, 2012; Regier, Kemp, & Kay, 2015; Xu & Regier, 2014; Xu, Regier, & Malt, 2016; Xu, Liu, & Regier, 2020).

The present work extends this analysis to *indefinite pronouns*, a domain of *function words*. Examples of indefinite pronouns in English are expressions such as *someone, something, anyone, anything, no one, nothing*.^1^ Syntactic, semantic, and typological properties of indefinite pronouns have been extensively studied by comparative linguists: Haspelmath's (1997) seminal work on indefinite pronouns includes a dataset on their meaning and distribution in 40 languages. We will rely heavily on this dataset in our study.

There are at least two reasons to pursue the extension of the simplicity/informativeness trade‐off analysis to indefinite pronouns. First, it would strengthen the case that the simplicity/informativeness trade‐off shapes both content and function word categories in language. In doing so, our work aligns with several concurrent studies exploring the simplicity/informativeness trade‐off in functional vocabulary (Mollica et al., 2021; Steinert‐Threlkeld, 2019, 2021; Uegaki, 2022; Zaslavsky et al., 2021).

Second, this analysis would help make progress on two related questions: (i) what explains the variation among natural languages, and (ii) what explains *linguistic universals*, which are properties common to all (or nearly all) human languages. More concretely, in relation to (i), a striking finding of Haspelmath's typological research is that no two languages in his corpus have the same systems of indefinite pronouns: in other words, it is not possible to establish a one‐to‐one mapping between any two languages' indefinite pronouns' meaning and distribution. Strikingly, and in relation to (ii), this diversity is constrained in important ways, which Haspelmath formulates as *implicational linguistic universals*. In this work, we ask whether natural languages are different (near‐)optimal solutions to the simplicity/informativeness trade‐off problem, which would explain some of the variation observed among natural languages' indefinite pronouns systems, as well as whether the simplicity/informativeness trade‐off may explain linguistic universals in the domain of indefinite pronouns.

The paper is organized as follows. First, we set the background for our analysis by (i) describing the meaning space of indefinite pronouns, (ii) detailing how Haspelmath's (1997) dataset is used in our research, and (iii) explaining how simplicity and informativeness of languages are measured. We then report the results of two computational experiments. The first experiment demonstrates that natural languages' indefinite pronoun systems are (near‐)optimal in how they trade off simplicity and informativeness. This holds even when we control for two properties of natural languages: the degree of overlap in indefinite pronouns' meanings in a language and the degree of coverage of the meaning space by indefinite pronouns in a language. The second experiment demonstrates that Haspelmath's implicational universals may be explained by the simplicity/informativeness trade‐off optimization. We proceed to replicate the two experiments in three additional settings pertaining to how simplicity and informativeness are measured. Finally, we discuss the implications of the results.

## 2 Indefinite pronouns: Meaning space and Haspelmath's universals

We first explain the space of possible meanings expressed by indefinite pronouns: what are the meanings that interlocutors may want to communicate by an indefinite pronoun?

**Fig. 1:**
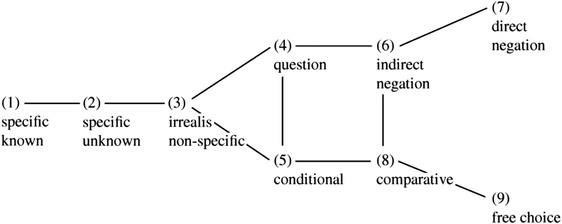
Haspelmath's (1997) map of functions of indefinites.

Haspelmath (1997) describes each indefinite pronoun in each language in his dataset in terms of which *functions* it can take. These functions are depicted on a “map” in Fig. 1. Some of them are meaning‐driven (functions 1, 2, 3, and 9 in Fig. 1, i.e., specific known, specific unknown, nonspecific, and free choice), and others are driven by syntactic distribution (functions 4, 5, 6, 7, and 8 in Fig. 1, i.e., question, conditional, comparative, indirect negation, and direct negation).

Haspelmath's functions thus mix meaning and syntactic distribution, and as such cannot be (all) taken as components of the meaning space of indefinite pronouns. To focus purely on meaning, we introduce *semantic flavors* and translate Haspelmath's syntactic functions into them. More specifically, Haspelmath's syntactic functions relate to two well‐studied categories of indefinite pronouns: *negative polarity indefinites* and *negative indefinites* (Bernini & Ramat, 1996; Fauconnier, 1975; Haspelmath, 1997; Ladusaw, 1979; Penka, 2011). In the present work, we abstract away from certain differences in the syntactic distribution of different negative polarity indefinites, and likewise, of different negative indefinites. We thus assume that the meaning space that a system of indefinite pronouns has to cover consists of the following six semantic flavors, which are described informally and illustrated with an example in (1)–(6):

(1) ***Specific known flavor*** [the indefinite pronoun refers to a specific individual that the interlocutors can uniquely identify]:
*Someone* managed to mess this up — we all know who!
(2) ***Specific unknown flavor*** [the indefinite pronoun refers to a specific individual that the interlocutors cannot uniquely identify]:
  I heard that *someone* failed, but I don't know who.
(3) ***Nonspecific flavor*** [the indefinite pronoun is interpreted as an existential quantifier over some domain of possible referents, not referring to a specific individual]:
  You should probably talk to *someone* else about this too.
(4) ***Negative polarity flavor*** [the indefinite pronoun is interpreted as an existential quantifier over a widened domain of possible referents]:
  Less than three companies hired *anyone* this year.
(5) ***Free choice flavor*** [the indefinite pronoun is interpreted as a wide‐scope universal quantifier over some domain of possible referents]:
  You can hire almost *anyone* here: most of them great.
(6) ***Negative indefinite flavor*** [the indefinite pronoun is interpreted as a negated existential quantifier over some domain of possible referents]:
  Who went to the party? *No one*.

We indexed each indefinite pronoun in Haspelmath's (1997) corpus with the flavors it can convey as follows:

- Functions 1, 2, 3, and 9 correspond to flavors (1), (2), (3), and (5), respectively.
- To decide whether an indefinite pronoun can convey flavor (6), we collected data on whether indefinite pronouns in languages of Haspelmath's corpus can be interpreted as negated existentials, relying in most cases on occurrence in negative fragment answers, which is a test for negative indefinites (Bernini & Ramat, 1996).^2^
- Functions 4, 5, 6, 7, and 8 code for syntactic environments in which indefinites with negative polarity flavor are acceptable; however, indefinites with other flavors may also be acceptable in these environments. Because of this, if an indefinite pronoun can take some of these functions, we will only conclude that the indefinite can convey the negative polarity flavor if we have independent evidence that the indefinite in question cannot convey other flavors which would be acceptable in those environments. The criterion we employ is the following. If (a) an item can take functions 4 and/or 6 but not function 3; or (b) it can take function 8 in combination with 4 and/or 6, or it can take function 8 but cannot take function 9; or (c) it can take function 7 but not function 3 and cannot be interpreted as a negated existential; or (d) it can take function 5, but not functions 3 and 9, then it has flavor (d). The motivation behind this complicated disjunctive criterion for negative polarity flavor is as follows. (a) Both indefinites with nonspecific flavor and indefinites with negative polarity flavor can be used in questions and under the scope of negation, hence, we can only conclude from functions 4 and 6 that an indefinite has a negative polarity flavor if we know independently that it cannot get nonspecific flavor. (b) Indefinite pronouns with function 8 might have either negative polarity or free choice flavor: items with negative polarity but not free choice flavor, such as English *ever*, are acceptable in comparatives, but so are instances of *any* modified by *almost*, and modification by *almost* is commonly taken as evidence for the free choice interpretation of *any* (cf. Aloni & Roelofsen, 2014; Heim, 2006). If an item can take function 8 in combination with functions 4 and/or 6, with the latter two environments precluding the free choice interpretation of indefinites (*any* cannot be modified by *almost* in those environments which evidences that free choice flavor is not available in those environments), we may conclude that the indefinite pronoun can have the negative polarity flavor. Similarly, if an indefinite pronoun takes function 8 but not function 9, we may conclude that it cannot convey the free choice flavor and thus must be conveying the negative polarity flavor. (c) As all negative indefinites are indexed with function 7 by Haspelmath, we only rely on function 7 as revealing the negative polarity flavor if the indefinite in question cannot be interpreted as negated existential, and if it cannot get nonspecific flavor more generally, for the same reasons as above. (d) Finally, in the antecedents of conditionals (function 5), we may find indefinites with negative polarity, nonspecific, and free choice flavor; we can thus conclude from function 5 that an indefinite has a negative polarity flavor if we know independently that it cannot get nonspecific or free choice flavor.

The data on indefinite pronouns presented in Haspelmath, 1997 show that languages differ greatly in how they cover the meaning space with their lexical items. Haspelmath, however, established that this variation is constrained. In particular, the map in Fig. 1 is devised to represent Haspelmath's findings about the constraints on the variation of indefinite pronoun systems: any indefinite pronoun in any language can only take functions which form a connected area on the map in Fig. 1. For example, if an indefinite pronoun can take functions 2 and 4, it is also able to take function 3. As another example, if an indefinite pronoun can take functions 6 and 9, it is also able to take function 8. These statements, of the form *‘If an indefinite pronoun can take functions X and Y, it is also able to take function Z,'* are called *Haspelmath's (implicational) universals*.

As we focus on semantic flavors rather than functions for reasons explained above, we note that it is relatively straightforward to ‘translate’ Haspelmath's universals from functions to flavors in the same principled way as established above (this allows us to think of Haspelmath's universals as constraints on the meanings of indefinite pronouns). Here are two examples of such ‘translations’ of universals. (i) If an item can convey the specific unknown and the negative polarity flavor, it can convey the nonspecific flavor. (ii) If an item can convey the specific known and the nonspecific flavor, it can also convey the specific unknown flavor.

We conclude this section by providing two examples of indefinite pronoun systems (focusing on the ontological category person) from the corpus by Haspelmath (1997): English in Table 1 and Russian in Table 2. Observe that, while these two indefinite pronoun systems are very different, their items obey Haspelmath's universals.

**Table 1:** English indefinite pronouns and the flavors they convey

Indefinite pronoun	Flavors
someone	specific known, specific unknown, nonspecific
anyone	negative polarity, free choice
no one	negative indefinite

**Table 2:** Russian indefinite pronouns and the flavors they convey

Indefinite pronoun	Flavors
kto‐to	specific unknown, nonspecific
kto‐nibud'	nonspecific
kto‐libo	nonspecific, negative polarity
nikto	negative indefinite
koe‐kto	specific known
kto by to ni bylo	negative polarity
kto ugodno	free choice

## 3 Measuring simplicity and informativeness

Before we introduce the relevant measures, a terminological note is in order. We will, henceforth, in line with the previous work, talk about *complexity* as the opposite of simplicity, and of *communicative cost* as the opposite of informativeness. One may thus equivalently speak of languages striving to maximize simplicity and informativeness, or striving to minimize complexity and communicative cost, that is, of simplicity/informativeness trade‐off or complexity/communicative cost trade‐off. For present purposes, we will define measures of complexity, informativeness, and communicative cost, with communicative cost being a decreasing function of informativeness, and analyze the trade‐off between complexity and communicative cost.

### 3.1 Complexity

Our measure of complexity relies on featural make‐up of indefinite pronouns. For this measure, we will again build on Haspelmath's work. Haspelmath (1997) (chapter 5) proposes that there are five binary features indefinite items can carry: known to the speaker (K), specific (S), scalar endpoint (SE), scale reversal (R), and in the scope of negation (N). Haspelmath further assumes that the feature R (+ or −) requires the indefinite pronoun to carry $SE^{+}$ feature.

Let us review briefly what these features stand for in Haspelmath's work. The features K and S are relatively transparent. S relates to the semantic notion of specificity, that is, whether the speaker has a specific referent in mind for the indefinite pronoun. K relates to whether or not the referent is known to the speaker. As for SE, Haspelmath (1997) motivates it from Fauconnier's work on negative polarity and free choice indefinites. In short, negative polarity and free choice indefinites evoke a pragmatic scale of alternatives ordered by likelihood, much like scalar focus particle *even*. For instance, *Mary can even solve the hardest problem* evokes alternatives, such as *Mary can solve the medium problem* and *Mary can solve the easiest problem*, which are ordered by likelihood as follows: *the hardest problem* < *the medium problem* < *the easiest problem*. According to this account, similar alternatives are activated by sentences with negative polarity and free choice indefinites, as in *Mary can solve any problem*, where *any* is the free choice indefinite. Furthermore, according to this account, negative polarity and free choice indefinites associate with the lowest (least likely) endpoint of the scale (hence, the name of the feature ‘scalar endpoint') — *Mary can solve any problem* is interpreted as *Mary can solve even the hardest problem* (Fauconnier, 1975). The R (“reversal”) feature relates to scale reversing contexts. Such contexts reverse the order by likelihood of alternatives on the pragmatic scale. For instance, we have seen that in contexts which are not scale reversing, the order by likelihood of alternatives may be *the hardest problem* < *the medium problem* < *the easiest problem*; in scale reversing contexts that order would be *the easiest problem* < *the medium problem* < *the hardest problem*. An example of a scale reversing context is the scope of negation, for example, *Mary didn't even solve the easiest problem*. Negative polarity indefinites appear in scale reversing contexts: *Mary didn't solve any problem*, where *any* is the negative polarity indefinite, is interpreted as *Mary didn't even solve the easiest problem*. The R feature reflects that negative polarity indefinites should associate with the lowest point on the scale in the scale reversing contexts ($R^{+}$), while free choice indefinites associate with the lowest point on the scale in the contexts which are not scale reversing ($R^{-}$), as illustrated above. Finally, the N feature relates to whether the indefinite pronoun expresses negation.

Haspelmath assumes that each of these five binary features characterizes a subset of functions of indefinite pronouns (cf. Haspelmath, 1997 for details). In the continuation, we assume the five binary features to characterize sets of semantic flavors as follows:^3^

- $K^{+}$: specific known
$K^{-}$: specific unknown, nonspecific, negative polarity, free choice, negative indefinite
- $S^{+}$: specific known, specific unknown
$S^{-}$: nonspecific, negative polarity, free choice, negative indefinite
- $SE^{+}$: negative polarity, free choice, negative indefinite
$SE^{-}$: specific known, specific unknown, nonspecific
- $R^{+}$: negative polarity, negative indefinite
$R^{-}$: free choice
- $N^{+}$: negative indefinite
$N^{-}$: specific known, specific unknown, nonspecific, negative polarity, free choice

Haspelmath does not provide a general recipe for how to generate the featural make‐up of an indefinite from the combination of functions that it might be able to take. We provide here one such recipe which is simple yet general enough to enable us to define any item in terms of its feature content based on the semantic flavors it can convey. Let us treat features as sets of flavors that they characterize, and the combination of features to correspond to set‐theoretic operations of intersection or union. We then define the featural make‐up of the indefinite pronoun to be *the shortest formula*—in a language whose primitives are the five binary features listed above and the set‐theoretic operations of union and intersection—that would correspond exactly to the flavor(s) an item can convey. For instance, in this language, the two formulae ‘$K^{+}$’ and ‘$K^{+}\cap S^{+}$’ amount to the same set of flavors, that is, {specific known}. However, because ‘$K^{+}$’ is a shorter formula than ‘$K^{+}\cap S^{+}$,’ we consider the featural make‐up of an indefinite pronoun that conveys only the specific known flavor to be ‘$K^{+}$’ and not ‘$K^{+}\cap S^{+}$.'

We measure the *complexity of an item* *c(i)* as the number of features in its featural make‐up (repetitions of the same features are counted as well). For instance, the featural make‐up of an item which can convey only the specific unknown flavor would be ‘$S^{+}\cap K^{-}$,’ and its complexity would thus be 2. This measure relates to the length of the formula in terms of the number of primitives: as we are assuming that the features can be combined by binary set‐theoretic operations, if there are n features in a formula, there will be n−1 set‐theoretic operators.

Our measure of *complexity of a language* *Complexity(L)* is defined as the sum of complexity measures of each item in the language (cf. (7)).

(7) **Complexity of a language**:
$$Complexity\left(L\right)=\sum_{i\in L}c\left(i\right)$$

Let us apply the notions developed in this section to the examples of English and Russian (cf. Tables 1 and 2). The feature formula and complexity of each indefinite pronoun in English and Russian is in Tables 3 and 4, respectively.

**Table 3:** English indefinite pronouns, the flavors they convey, their feature formulae, and their complexities

Indefinite pronoun	Flavors	Feature formula	c(i)
someone	specific known, specific unknown, nonspecific	$SE^{-}$	1
anyone	negative polarity, free choice	$SE^{+}\cap N^{-}$	2
no one	negative indefinite	$N^{+}$	1

**Table 4:** Russian indefinite pronouns, the flavors they convey, their feature formulae, and their complexities

Indefinite pronoun	Flavors	Feature formula	c(i)
kto‐to	specific unknown, nonspecific	$K^{-}\cap SE^{-}$	2
kto‐nibud'	nonspecific	$S^{-}\cap SE^{-}$	2
kto‐libo	nonspecific, negative polarity	$\left(S^{-}\cap SE^{-}\right)\cup \left(\left(SE^{+}\cap R^{+}\right)\cap N^{-}\right)$	5
nikto	negative indefinite	$N^{+}$	1
koe‐kto	specific known	$K^{+}$	1
kto by to ni bylo	negative polarity	$\left(SE^{+}\cap R^{+}\right)\cap N^{-}$	3
kto ugodno	free choice	$SE^{+}\cap R^{-}$	2

To compute the complexity of English and Russian indefinite pronoun systems, we can simply sum the column c(i) in Tables 3 and 4, respectively (cf. (7)). The complexity of English is 4, and the complexity of Russian is 16 — Russian indefinite pronoun system is thus more complex than English indefinite pronoun system.

### 3.2 Informativeness and communicative cost

Our measure of informativeness is rooted in the notion of successful communication. The communication unfolds as follows. A speaker has a semantic flavor f from the set of flavors F in mind that they want to communicate to a listener by using an indefinite pronoun i from their language L; the listener tries to guess upon hearing i which flavor the speaker intended (Kemp, Xu, & Regier, 2018; Skyrms, 2010; Steinert‐Threlkeld, 2019, 2021). We assume a simplified communicative scenario between a literal speaker and a literal listener who do not engage in pragmatic reasoning about each other's communicative intentions: these agents’ production and interpretation of indefinite pronouns follow solely from their semantics.^4^ The literal speaker's probability to use i to communicate f, $P_{S-lit}\left(i|f\right)$ is defined in (8). The literal listener's probability to guess f upon hearing i, $P_{L-lit}\left(f|i\right)$ is defined in (9). [[·]] is the interpretation function; [[i]](f)=1 iff f∈i (iff f is a possible interpretation of i). For instance, in English, [[anyone]](negativepolarityflavor)=1, while [[anyone]](specificknownflavor)=0 (cf. Table 1). We further note that the literal listener is closely related to $L_{0}$ communicative agent from the basic rational speech act (RSA) model (Frank & Goodman, 2012) (the basic RSA model is discussed in greater detail in Section 6).

(8) **Literal speaker**:
$$P_{S-\mathrm{lit}}\left(i|f\right)=\frac{\left[[i]](f\right)}{\sum_{i^{\prime }\in L}\left[\left[i^{\prime }\right]\right]\left(f\right)}$$
(9) **Literal listener**:
$$P_{L-\mathrm{lit}}\left(f|i\right)=\frac{\left[[i]](f\right)}{\sum_{f^{\prime }\in F}\left[\left[i\right]\right]\left(f^{\prime }\right)}$$

For instance, according to (8), if the literal speaker of Russian wanted to communicate the negative polarity flavor with an indefinite pronoun, the probability that they would use *kto‐libo* is 0.5 (because they have a choice between *kto‐libo* and *kto by to ni bylo* to communicate the negative polarity flavor, cf. Table 2). According to (9), if the literal listener of Russian hears the indefinite pronoun *kto‐to*, the probability that they would interpret it with the specific unknown flavor is 0.5 (because *kto‐to* may be used to communicate the specific unknown or the nonspecific flavor, cf. Table 2).

The informativeness of a language is defined in (10). It corresponds to the probability that the communication will be successful given the need to communicate different flavors, reflected in the prior over flavors from the set of flavors F, as well as the speaker's and listener's probability.

(10) **Informativeness of a language**:
$$I\left(L\right)=\sum_{f\in F}\sum_{i\in L}P\left(f\right)P_{L-lit}\left(f|i\right)P_{S-lit}\left(i|f\right)$$

The prior over flavors is estimated from the corpus in Beekhuizen, Watson, and Stevenson, 2017 in which indefinite pronouns in English are annotated for Haspelmath's functions. We focused on indefinite pronouns for the ontological category person, as our later analyses focus on this ontological category as well. In English, *any‐* indefinite pronouns (e.g., *anyone*) can have the negative polarity and the free choice flavor (the functions they can take are 4, 5, 6, 7, 8, and 9); *some‐* indefinite pronouns (e.g., *someone*) can have the specific known, specific unknown, and nonspecific flavor (the functions they can take are 1, 2, 3, 4, and 5); *no‐* indefinite pronouns (e.g., *no one*) can have the negative indefinite flavor (the function they can take is 7) (cf. Table 1). In order to ‘translate’ the annotated functions of the English indefinite pronouns from Beekhuizen et al.'s (2017) corpus, we proceeded as follows. If an indefinite pronoun was *anyone* or *anybody* and it was annotated to have one of the functions 4, 6, and 7 (*question, indirect negation, direct negation*), we indexed it as having the *negative polarity flavor*. If an indefinite pronoun was *anyone* or *anybody* and it was annotated to have one of the functions 5 and 8 (*conditional, comparative*), we indexed it as having the *negative polarity flavor* half of the time, and as having the *free choice flavor* half of the time (this is because these environments allow for both *negative polarity* and *free choice* flavor; we have no data on the rate of each of these flavors in these two environments and thus assume them to be equally frequent). If an indefinite pronoun was *anyone* or *anybody* and it was annotated to have the function 9 (*free choice*), we indexed it as having the *free choice flavor*. If an indefinite pronoun was *no one, nobody*, we indexed it as having the *negative indefinite flavor*. Beekhuizen et al. (2017) did not distinguish between functions 1 and 2 (*specific known* and *specific unknown*) for indefinite pronouns *someone, somebody*, and annotated the indefinite pronouns which had one of those two functions with *specific* only. For lack of evidence to the contrary, we assume that among the indefinite pronouns annotated with *specific* by Beekhuizen et al. (2017), those with *specific known* and those with *specific unknown* flavor are equally frequent. If an indefinite pronoun was *someone, somebody* and it was annotated to have functions other than *specific*, we indexed it as having the *nonspecific flavor*. The estimated priors over flavors are provided in Table 5.

**Table 5:** Prior probability distribution over flavors, as estimated from the corpus in Beekhuizen et al., 2017

Semantic flavor	Prior probability
specific known	0.08
specific unknown	0.08
nonspecific	0.26
negative polarity	0.33
free choice	0.1
negative indefinite	0.15

The *communicative cost* of a language should be a decreasing function of the informativeness of the language; we define the communicative cost of a language L in (11). This means that maximizing informativeness of a language is equivalent to minimizing communicative cost.

(11) **Communicative cost of a language**:
$$Cost\left(L\right)=\frac{1}{I(L)}$$

Going back to the examples of English and Russian, if we compute the communicative cost of the two languages according to (10) and (11), we will find that the communicative cost of English is 1.98, and the communicative cost of Russian is 1.27. Russian indefinite pronoun system thus has a lower communicative cost than English indefinite pronoun system. This makes sense from the perspective of the simplicity/informativeness trade‐off: while Russian is more complex, it has a lower communicative cost than English, that is, it has chosen to trade some simplicity for some informativeness.

## 4 Experiment 1

In Experiment 1,^5^ we address the question of whether natural languages optimize the simplicity/informativeness trade‐off in the domain of indefinite pronouns.

For our purposes, a language is a set of indefinite pronouns for the ontological category person (e.g., *someone, anyone, no one*).^6^ We evaluate the optimality of natural languages by measuring how distant they are from the Pareto frontier in comparison to artificially generated languages. The Pareto frontier consists of Pareto optimal languages; a language is (Pareto) optimal if there is no other language that has both lower complexity and lower communicative cost. The idea is that, if natural languages optimize the simplicity/informativeness trade‐off, they should be more optimal than artificially generated languages, and being more optimal means being closer to the set of optimal languages (the Pareto frontier).

To evaluate this, we artificially generated 10,000 languages, which could have between 1 and 7 indefinite pronouns (7 is the maximum number of indefinite pronouns that any natural language has in Haspelmath's corpus). Each indefinite pronoun in each artificial language was randomly assigned one of the 63 logically possible combinations of flavors ($2^{6}-1$ combinations, excluding an indefinite pronoun that does not convey any of the six flavors).

Random assignment of the 63 logically possible combinations of flavors to items of artificial languages resulted in artificial languages having more items with overlapping meanings and more semantic gaps than natural languages. These two properties put artificial languages at a disadvantage compared to natural languages with respect to simplicity/informativeness trade‐off for an uninteresting reason. Because of this, before we compare natural and artificial languages with respect to simplicity/informativeness trade‐off, we control for this difference as follows.

The artificial languages were matched to natural languages for *the degree of overlap*, defined in (12). The degree of overlap captures how many different indefinite pronouns in a language can be used to express the same flavor: if the indefinite pronouns in the language have more overlapping meanings, the degree will be higher. Matching ensured that the average degree of overlap among natural languages (call it $\bar{\mathrm{nat}\_\mathrm{overlap}}$) is similar to the average degree of overlap among artificial languages.

(12) **Degree of overlap**:
$$Overlap\left(L\right)=\sum_{f\in F}\left(|\left\{i\in L:f\in i\right\}|-1\right)$$

Furthermore, the artificial languages were matched to natural languages for *the degree of coverage*, defined in (13). The degree of coverage measures how many flavors can be expressed by indefinite pronouns in a language. As before, matching ensured that the average degree of coverage among natural languages (call it $\bar{\mathrm{nat}\_\mathrm{coverage}}$) is similar to the average degree of coverage among artificial languages.

(13) **Degree of coverage**:
Coverage(L)=|{f∈F:∃i∈L.f∈i}|

The matching procedure was solving the optimization problem in (14), which is an integer programming problem (Schrijver, 1998). The objective is to find a maximal subset of the 10,000 artificially generated languages under the following constraints: (i) their average degree of overlap is within $\bar{\mathrm{nat}\_\mathrm{overlap}}$ ·(1+/−ε); and (ii) their average degree of coverage is within $\bar{\mathrm{nat}\_\mathrm{coverage}}$ ·(1+/−ε), with ε=0.01. The procedure was implemented in R (R Core Team, 2020) using *ompr* package for modeling mixed integer linear programs (Schumacher, 2017) and *glpk* solver (Makhorin, 2008). Due to computational constraints, the time to run the algorithm was limited to 24 h, after which the best solution at that time was collected (note that this means that the collected solution is not necessarily the optimal solution, i.e., there may exist a larger subset of artificial languages, which falls within constraints (i) and (ii), but which was not found within the 24 h time limit).

(14)  
$$\mathrm{maximize}\sum_{k=1}^{10000}x_{k}$$
  subject to
$$\sum_{k=1}^{10000}x_{k}\cdot {\text{overlap}}_{k}\leq \bar{\mathrm{nat}\_\mathrm{overlap}}\cdot \left(1+\varepsilon \right)\cdot \sum_{k=1}^{10000}x_{k}$$
$$\sum_{k=1}^{10000}x_{k}\cdot {\text{overlap}}_{k}\geq \bar{\mathrm{nat}\_\mathrm{overlap}}\cdot \left(1-\varepsilon \right)\cdot \sum_{k=1}^{10000}x_{k}$$
$$\sum_{k=1}^{10000}x_{k}\cdot {\text{coverage}}_{k}\leq \bar{\mathrm{nat}\_\mathrm{coverage}}\cdot \left(1+\varepsilon \right)\cdot \sum_{k=1}^{10000}x_{k}$$
$$\sum_{k=1}^{10000}x_{k}\cdot {\text{coverage}}_{k}\geq \bar{\mathrm{nat}\_\mathrm{coverage}}\cdot \left(1-\varepsilon \right)\cdot \sum_{k=1}^{10000}x_{k}$$
  where
$$\forall k\in \left\{1,\cdots ,10000\right\},x_{k}=\left\{\begin{array}{cc} 1 & \text{if}\;\text{the}\;\text{artificial}\;\text{language}\;k\;\text{is}\;\text{in}\;\text{the}\;\text{matched}\;\text{set}\\ 0 & \text{otherwise} \end{array}\right.$$
  and
$${\text{overlap}}_{k}\;\text{is}\;\text{the}\;\text{degree}\;\text{of}\;\text{overlap}\;\text{of}\;\text{the}\;\text{artificial}\;\text{language}\;k,$$
$${\text{coverage}}_{k}\;\text{is}\;\text{the}\;\text{degree}\;\text{of}\;\text{coverage}\;\text{of}\;\text{the}\;\text{artificial}\;\text{language}\;k,$$
$$\bar{\mathrm{nat}\_\mathrm{overlap}}\;\text{is}\;\text{the}\;\text{average}\;\text{degree}\;\text{of}\;\text{overlap}\;\text{among}\;\text{natural}\;\text{languages},$$
$$\bar{\mathrm{nat}\_\mathrm{coverage}}\;\text{is}\;\text{the}\;\text{average}\;\text{degree}\;\text{of}\;\text{coverage}\;\text{among}\;\text{natural}\;\text{languages},$$
ε=0.01

After matching for overlap and coverage, 479 artificial languages remained for comparison to natural languages. After matching, mean degree of overlap of both natural and artificial is 0.68 (that of artificial was 7.1 pre‐matching), and mean degree of coverage of natural languages is 5.58, and that of artificial languages is 5.52 (that of artificial was 5.2 pre‐matching).

Controlling for overlap makes sure that, if natural languages are superior to artificial languages in terms of the simplicity/informativeness trade‐off, this is not simply due to artificial languages having more words whose meanings overlap. In addition to this, controlling for coverage makes sure that, if natural languages are superior to artificial languages in terms of the simplicity/informativeness trade‐off, this is not simply due to artificial languages having more semantic gaps.

**Fig. 2:**
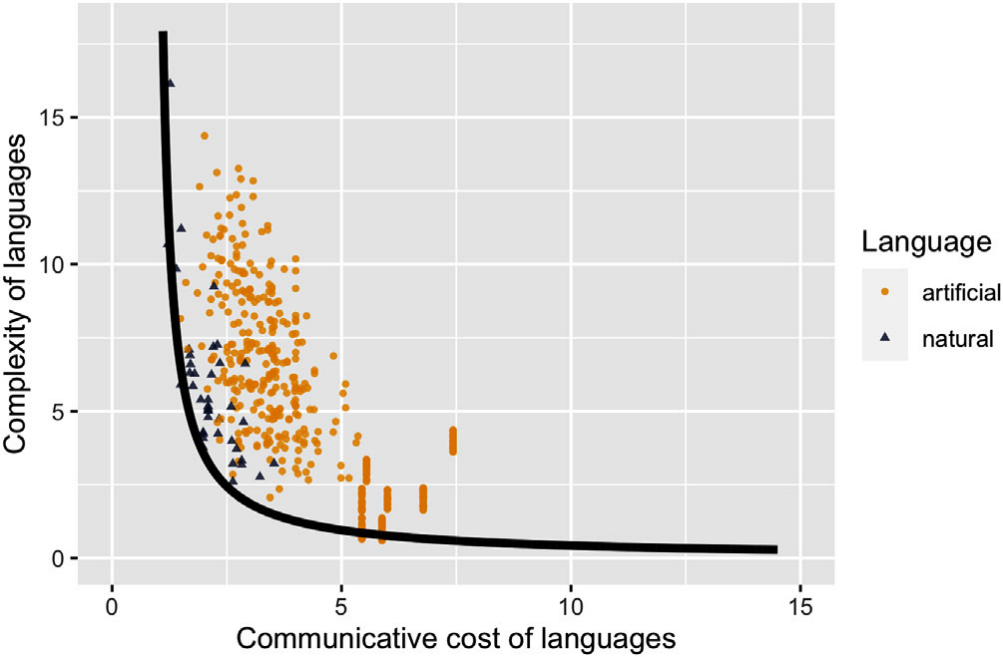
Experiment 1: Complexity and communicative cost of 40 natural and 479 artificial languages (natural and artificial languages are matched for the degree of overlap and coverage).

The artificially generated languages serve to map the space of possibilities for indefinite pronoun systems whose degrees of overlap and coverage are comparable to those of natural languages. We compute complexity and communicative cost of natural and (matched) artificial languages of Experiment 1, and plot them in Fig. 2.

We follow Steinert‐Threlkeld (2019) in using an evolutionary algorithm to estimate the Pareto frontier. The algorithm works as follows. First, the generation 0 is generated, which consists of 2000 randomly generated languages. The dominant languages (those for which there is no language which has both lower complexity and lower communicative cost) each gives rise to an equal number of offspring languages, which are obtained via a small number of mutations (between 1 and 3; these mutations included removing an item, adding an item, and interchanging an item) from dominant languages. The dominant languages from generation 0 together with their offspring languages constitute generation 1. This process is repeated for 100 generations. Finally, the dominant languages are selected from the union of the last generation, the 40 natural languages, and the matched artificial languages from Experiment 1. A curve in (15) is then fitted to the dominant languages to estimate the Pareto frontier. The estimated Pareto frontier is plotted as the black curve in Fig. 2.

(15)  
$$Complexity\left(L\right)∼\frac{a}{b+Cost(L)}$$

As can be seen in Fig. 2, most of the 40 natural languages lie near this Pareto frontier, suggesting that they are optimizing the simplicity/informativeness trade‐off. This is further supported by the comparison of distances from the Pareto frontier of natural and artificial languages. For each language, we compute its distance from the Pareto frontier as the minimum Euclidean distance between the language and a point on the Pareto frontier. We find that natural languages are significantly closer to the Pareto frontier than artificial languages ($M_{1}=0.48$, $M_{2}=1.55$, t(82.8)=−16, p<.001). Interestingly, focusing on where natural languages lie with respect to the Pareto frontier in Fig. 2, we see that while most natural languages lie in the lower left corner of the plot, with low communicative cost and low complexity, there is quite some variability in terms of what the closest point on the Pareto frontier for each of the natural languages would be. This suggests that some of the diversity that can be observed in the indefinite pronoun systems across languages is due to languages approaching different optimal solutions to the trade‐off problem (cf. also Kemp et al., 2018). This can be illustrated by our examples of English and Russian. Recall that the complexity of English is 4 and the complexity of Russian is 16, while the communicative cost of English is 1.98 and the communicative cost of Russian is 1.27 (cf. Sections 3.1 and 3.2). These two languages thus solve the simplicity/informativeness trade‐off in a different way, approaching different points on the Pareto frontier.

Finally, it is interesting to notice another property of the data. The 40 natural languages lie along the region of the Pareto frontier where communicative cost is relatively low (up to about 4). If this is true of natural languages’ indefinite pronoun systems in general, and not only of the 40 ones studied here, this entails that there is a baseline degree of informativeness below which natural languages are, in a sense, not willing to go as far as indefinite pronouns are concerned, even if it is still possible to achieve Pareto‐optimality with such lower levels of informativeness (the Pareto‐optimal very high cost languages tend to have a single highly ambiguous expression). This would, in turn, suggest that the simplicity/informativeness trade‐off optimization is not the only pressure shaping indefinite pronoun systems inventories: there seems to exist an additional pressure, namely, the pressure to have some baseline degree of informativeness, pushing toward certain regions of the Pareto frontier.

## 5 Experiment 2

Experiment 1 demonstrates that natural languages optimize the simplicity/informativeness trade‐off of their indefinite pronoun systems. The trade‐off optimization is thus likely to explain some of the variation between indefinite pronoun systems among natural languages, as well as some of their universal properties. Can the trade‐off explain some of Haspelmath's universals? To answer this question, in Experiment 2, we compare 5000 artificial languages which satisfy Haspelmath's universals, as originally stated, in terms of functions rather than flavors,^7^ to 5000 artificial languages which do not (henceforth, *Haspel‐ok* and *Not Haspel‐ok* languages, respectively). We do this comparison for the following reason: if the reason why natural languages satisfy Haspelmath's universals is because these help optimize the simplicity/informativeness trade‐off, then artificial languages which satisfy Haspelmath's universals should be more optimal than artificial languages which do not satisfy them. Languages of both groups had between 1 and 7 items, and *Not Haspel‐ok* languages were matched to *Haspel‐ok* languages for their degree of overlap and coverage. After matching, 2881 *Not Haspel‐ok* languages remain, with mean degree of overlap of *Haspel‐ok* languages 5.66 and that of *Not Haspel‐ok* 5.61 (that of *Not Haspel‐ok* was 7.4 prematching), and with mean degree of coverage of *Haspel‐ok* languages 4.58, and that of *Not Haspel‐ok* 4.63 (that of *Not Haspel‐ok* was 5.2 prematching).^8^ Each item in each *Haspel‐ok* language is sampled from a pool of all logically possible items which satisfy Haspelmath's universals (in terms of which of Haspelmath's functions they can take). On the other hand, each item in each *Not Haspel‐ok* language is sampled from a pool of all logically possible items (in terms of which of Haspelmath's functions they can take): items of *Not Haspel‐ok* languages thus may or may not conform to Haspelmath's universals. The Pareto frontier is estimated in the same way as in Experiment 1.

**Fig. 3:**
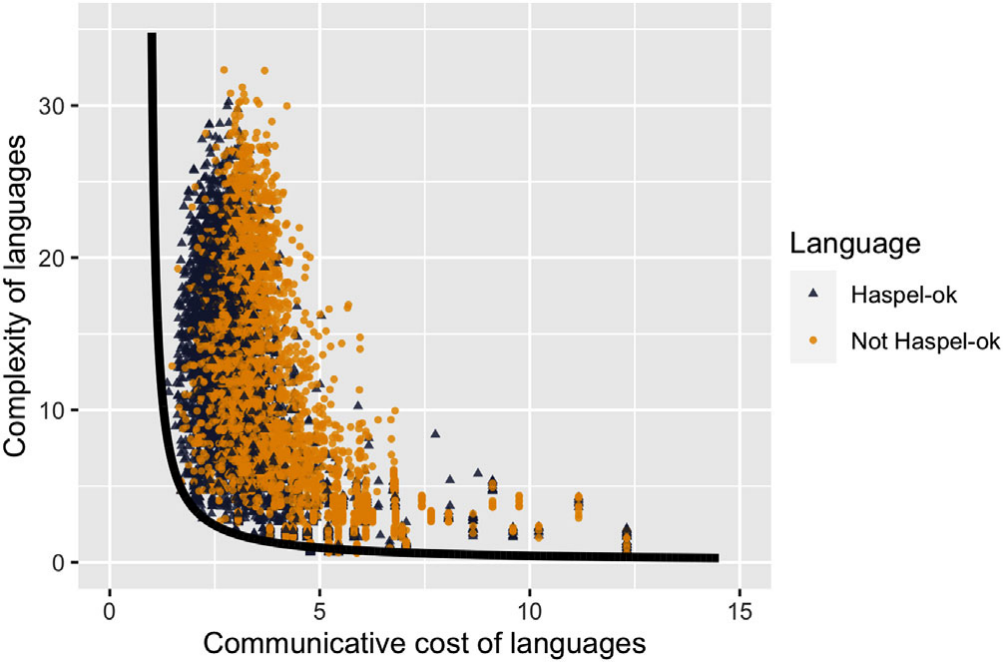
Experiment 2: Complexity and communicative cost of 5000 artificial languages which satisfy Haspelmath's universals and 2881 artificial languages which do not (*Haspel‐ok* and *Not Haspel‐ok* languages are matched for the degree of overlap and coverage).

In Fig. 3, we plot the complexity and communicative cost of *Haspel‐ok* and of (matched) *Not Haspel‐ok* languages, as well as the estimated Pareto frontier. Using the same measure of distance from Pareto frontier as in Experiment 1, we find that the *Haspel‐ok* languages are significantly closer to Pareto frontier than the *Not Haspel‐ok* languages ($M_{1}=1.61$, $M_{2}=2.26$, t(4819.4)=−34.3, p<.001). This demonstrates that languages which satisfy Haspelmath's universals are indeed better at trading simplicity and informativeness than languages which do not. Importantly, the results of Experiment 2 demonstrate that this holds in general, and not only for the 40 natural languages from Haspelmath's corpus.

Why is it the case that languages satisfying Haspelmath's universals trade more optimally between simplicity and informativeness? Fundamentally, Haspelmath's universals are descriptions of what kinds of ambiguities are allowed and what kinds of ambiguities are not allowed when it comes to flavors an indefinite pronoun may convey. What kind of ambiguities indefinite pronouns have in a given language influences the measure of informativeness of the language: some ambiguities are better to have than others as far as informativeness is concerned. For instance, a language which has an indefinite pronoun ambiguous between the specific known and the specific unknown flavor and nonambiguous indefinite pronouns for the remaining four flavors will have a higher degree of informativeness than a language which has an indefinite pronoun ambiguous between the nonspecific and the negative polarity flavor and nonambiguous indefinite pronouns for the remaining four flavors. The reason is that the prior probability to talk about specific known or specific unknown flavor is lower than the prior probability to talk about the nonspecific or negative polarity flavor. That languages satisfying Haspelmath's universals trade more optimally between simplicity and informativeness suggests that, on average, adding an indefinite pronoun whose complexity is n to a system of indefinite pronouns will be more advantageous for the system's informativeness if the ambiguity between multiple flavors of the indefinite pronoun is in line with Haspelmath's universals.

## 6 Alternative measures of simplicity and informativeness

The simplicity/informativeness trade‐off analyses depend heavily on the way simplicity and informativeness are measured (equivalently, complexity and communicative cost). We will now consider one alternative way of measuring complexity, and one alternative way of measuring informativeness and communicative cost, motivated below.

### 6.1 Alternative measure of complexity: Adding the set difference

Five binary features, as well as flavors they are compatible with, were introduced in Section 3.1. The featural make‐up of an indefinite pronoun was defined to be the shortest formula in a language whose primitives are the five binary features and the set‐theoretic operations of union and intersection.

Combining features via union and intersection suffices to generate all possible flavor combinations. It is, however, conceivable that there are other operations which are cognitively available for feature combination in addition to (or even instead of) union and intersection. We will explore one more option: that according to which union, intersection, and *set difference* are the available operations for feature combination.^9^

In other words, we will consider an alternative definition of the featural make‐up of an indefinite pronoun as the shortest formula in a language whose primitives are the five binary features and the set‐theoretic operations of union, intersection, and set difference. Complexity of an item and complexity of a language are defined as in Section 3.1, modulo the new approach to the featural make‐up of the indefinite pronouns.

We note, however, that the two options explored in this paper, (i) union and intersection; (ii) union, intersection, and set difference as operations for combining features, clearly do not exhaust the space of choices (one could explore adding set complement and/or symmetric difference in addition to or instead of the set difference, among others). The reason for exploring one additional option, that is, the option (ii), is simply to illustrate that, at present, we do not have a definite answer to the question of which operations are the cognitively available ones for feature combinations, but that one can nonetheless explore the robustness of the findings with respect to different candidate hypotheses for what these operations may be.

### 6.2 Alternative measure of informativeness and communicative cost: Pragmatic speakers and listeners

Our original measure of informativeness was rooted in the notion of successful communication between a literal speaker and a literal listener, who do not reason about each other's communicative intentions in producing or interpreting indefinite pronouns. It is, however, plausible that languages are optimizing for successful communication between more sophisticated communicative agents: henceforth, a pragmatic speaker and a pragmatic listener (cf. also Uegaki, 2022). In defining the pragmatic speaker and the pragmatic listener for indefinite pronouns use and interpretation, we rely heavily on the basic RSA model (Frank & Goodman, 2012): the pragmatic speaker and the pragmatic listener are closely related to $S_{1}$ and $L_{1}$ communicative agents from the basic RSA model.

The pragmatic speaker considers the utility of different utterances (indefinite pronouns) to communicate the meaning they have in mind, defined in (16): an indefinite pronoun i is useful at communicating some flavor f if the literal listener assigns a high probability to the flavor f after hearing i.^10^

(16) **Pragmatic speaker's utility**:
$$U_{S-\mathrm{prag}}\left(i;f\right)=\log P_{L-\mathrm{lit}}\left(f|i\right)$$

The pragmatic speaker acts rationally according to Bayesian decision theory by choosing different utterances (indefinite pronouns) in proportion to their utility: the pragmatic speaker's probability to use i to communicate f, $P_{S-prag}\left(i|f\right)$ is defined in (17). In other words, what (17) models is the following: the pragmatic speaker reasons about how the literal listener will interpret their utterances, and chooses an indefinite pronoun i to communicate a flavor f proportionally to how well i communicates f to the literal listener. The temperature parameter α in the definition of the pragmatic speaker in (17), which controls how rational the speaker's decision making is, is assumed to be 1: this is a typical choice when no strong assumptions about the speaker's rationality are being made. The pragmatic listener's probability to guess f upon hearing i, $P_{L-prag}\left(f|i\right)$ is defined in (18). What (18) models is the following: the pragmatic listener reasons about how the pragmatic speaker chooses their utterances and interprets an indefinite pronoun i as conveying f proportionally to the probability that the pragmatic speaker would use i to communicate f, together with the prior probability that f needs to be communicated.

(17) **Pragmatic speaker**:
$$P_{S-prag}\left(i|f\right)=\frac{exp(\alpha \cdot U_{S-prag}\left(i;f\right))}{\sum_{i^{\prime }\in L}exp\left(\alpha \cdot U_{S-prag}\left(i^{\prime };f\right)\right)}$$
(18) **Pragmatic listener**:
$$P_{L-prag}\left(f|i\right)=\frac{P_{S-prag}\left(i|f\right)\cdot P\left(f\right)}{\sum_{f^{\prime }\in F}P_{S-prag}\left(i|f^{\prime }\right)\cdot P\left(f^{\prime }\right)}$$

For instance, according to (17), if the pragmatic speaker of Russian wanted to communicate the negative polarity flavor with an indefinite pronoun, the probability that they would use *kto‐libo* is 0.33 (recall that the literal speaker's probability was 0.5, cf. Section 3.2). Conceptually, this decrease in probability is because the pragmatic speaker realizes that *kto by to ni bylo* is a better expression to use than *kto‐libo* to communicate the negative polarity flavor to the literal speaker (this is because *kto‐libo* can communicate the negative polarity or the nonspecific flavor, while *kto by to ni bylo* can only communicate the negative polarity flavor, cf. Table 2). According to (18), if the pragmatic listener of Russian hears the indefinite pronoun *kto‐to*, the probability that they would interpret it with specific unknown flavor is 0.55 (recall that the literal listener's probability was 0.5). Conceptually, this slight increase in probability is because the pragmatic listener realizes that the speaker has better options to communicate the nonspecific flavor, which is the other possible interpretation of *kto‐to*. The increase is only slight because the prior probability to communicate the nonspecific flavor is higher than the prior probability to communicate the specific unknown flavor (cf. Table 5).

With this, the revised definition of informativeness is as follows:

(19) **Informativeness of a language (pragmatic version)**:
$$I\left(L\right)=\sum_{f\in F}\sum_{i\in L}P\left(f\right)P_{L-prag}\left(f|i\right)P_{S-prag}\left(i|f\right)$$

The communicative cost of a language is defined as in Section 3.2, modulo the revised definition of informativeness.

### 6.3 Results

Experiments 1 and 2 were repeated with these new measures of complexity and communicative cost. We report here the results of these replications with the following settings: (i) the original measure of communicative cost (which we will refer to as *literal cost*) in combination with the new measure of complexity (which we will refer to as *3‐operators complexity*); (ii) the new measure of communicative cost (which we will refer to as *pragmatic cost*) in combination with the original measure of complexity (which we will refer to as *2‐operators complexity*); and (iii) pragmatic cost in combination with 3‐operators complexity.

The results replicate entirely in these three alternative settings.

#### Experiment 1‐i and Experiment 2‐i: Literal cost and 3‐operators complexity

The procedure for conducting Experiments 1‐i and 2‐i was identical to those for Experiments 1 and 2, respectively, modulo the following: while Experiments 1 and 2 employed 2‐operators complexity, Experiments 1‐i and 2‐i employed 3‐operators complexity.

**Fig. 4:**
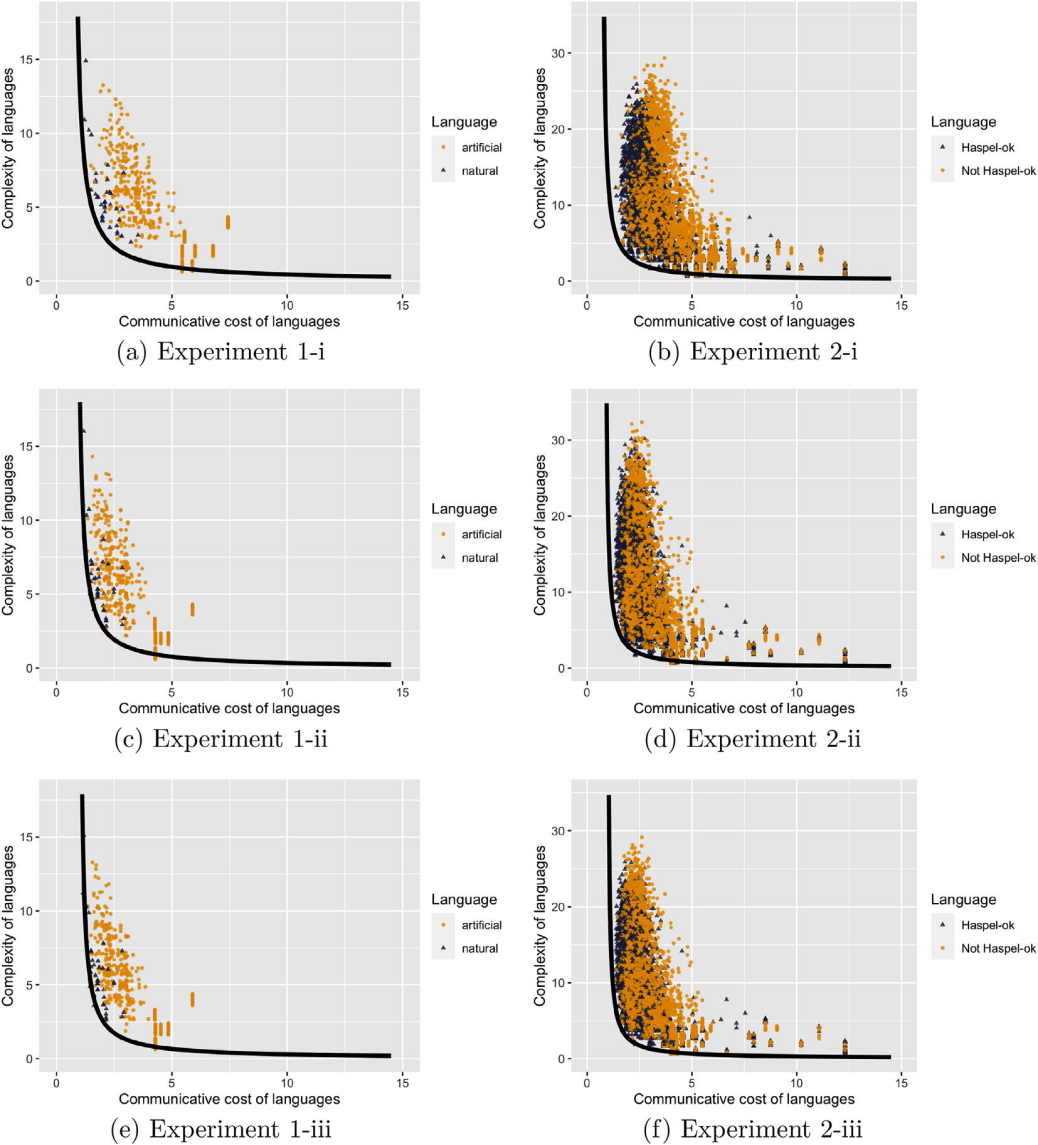
*Row 1*: Replications of Experiments 1 and 2 with settings: literal cost, 3‐operators complexity. *Row 2*: Replications of Experiments 1 and 2 with settings: pragmatic cost, 2‐operators complexity. *Row 3*: Replications of Experiments 1 and 2 with settings: pragmatic cost, 3‐operators complexity.

In Figs. 4a and b, we plot the results of Experiments 1‐i and 2‐i, respectively. In Experiment 1‐i, we find that the *natural* languages are significantly closer to Pareto frontier than the *artificial* languages ($M_{1}=0.6$, $M_{2}=1.6$, t(85.1)=−15.1, p<.001). In Experiment 2‐i, we find that the *Haspel‐ok* languages are significantly closer to Pareto frontier than the *Not Haspel‐ok* languages ($M_{1}=1.7$, $M_{2}=2.3$, t(4820.2)=−33.9, p<.001).

#### Experiment 1‐ii and Experiment 2‐ii: Pragmatic cost and 2‐operators complexity

The procedure for conducting Experiments 1‐ii and 2‐ii was identical to those for Experiments 1 and 2, respectively, modulo the following: while Experiments 1 and 2 employed literal cost, Experiments 1‐ii and 2‐ii employed pragmatic cost.

In Figs. 4c and d, we plot the results of Experiments 1‐ii and 2‐ii, respectively. In Experiment 1‐ii, we find that the *natural* languages are significantly closer to Pareto frontier than the *artificial* languages ($M_{1}=0.4$, $M_{2}=1.2$, t(73.9)=−12, p<.001). In Experiment 2‐ii, we find that the *Haspel‐ok* languages are significantly closer to Pareto frontier than the *Not Haspel‐ok* languages ($M_{1}=1.2$, $M_{2}=1.6$, t(4797.4)=−24.6, p<.001).

#### Experiment 1‐iii and Experiment 2‐iii: Pragmatic cost and 3‐operators complexity

The procedure for conducting Experiments 1‐iii and 2‐iii was identical to those for Experiments 1 and 2, respectively, modulo the following: while Experiments 1 and 2 employed literal cost, Experiments 1‐iii and 2‐iii employed pragmatic cost; while Experiments 1 and 2 employed 2‐operators complexity, Experiments 1‐iii and 2‐iii employed 3‐operators complexity.

In Figs. 4e and f, we plot the results of Experiments 1‐iii and 2‐iii, respectively. In Experiment 1‐iii, we find that the *natural* languages are significantly closer to Pareto frontier than the *artificial* languages ($M_{1}=0.4$, $M_{2}=1.2$, t(74.5)=−12.5, p<.001). In Experiment 2‐iii, we find that the *Haspel‐ok* languages are significantly closer to Pareto frontier than the *Not Haspel‐ok* languages ($M_{1}=1.1$, $M_{2}=1.5$, t(4731.3)=−25.8, p<.001).

## 7 Discussion

In this work, we have argued that the simplicity/informativeness trade‐off can explain how a language organizes its vocabulary to cover the meaning space of indefinite pronouns. In general, such work relies on three fundamental assumptions: (1) that we are indeed dealing with a proper category (i.e., that all and only the expressions we are considering form a (sub‐)system of a language); (2) that we have a good understanding of the meaning space that the system is covering; and (3) that we have a reasonable way to estimate the simplicity and informativeness of the system. These assumptions are sometimes left implicit in related work; we discuss them explicitly in relation to indefinite pronouns, and point out where they may be questioned and may evolve. We believe that the points we raise in this discussion may be of relevance for any work belonging to this paradigm.

### 7.1 Are indefinite pronouns a well‐defined category?

The question of whether the system of indefinite pronouns is optimized with respect to the simplicity/informativeness trade‐off presupposes that the expressions that fall under the label ‘indefinite pronoun’ indeed form a category across languages. One way to establish that something is a category is to provide a set of criteria that would separate members from nonmembers. Haspelmath (1997) defines indefinite pronouns as expressions (i) which are *grammatical*, that is, function words, that are syntactically mainly noun phrases, adverbial phrases, or adjectival phrases, and (ii) whose main semantic function is to express *indefinite reference*. Haspelmath, however, acknowledges that (ii) is too narrow to qualify as indefinite pronouns all and only the expressions that he lists under the term: for instance, the indefinite pronoun *nobody* arguably does not express indefinite reference, but rather conveys the nonexistence of a referent. A more general semantic criterion is thus needed for the category of indefinite pronouns to be well‐defined.

While it is beyond the scope of this work to attempt to identify such a more general semantic criterion, let us point out that much semantic work since Haspelmath has indeed argued in favor of close semantic connections between expressions that Haspelmath considers to be ‘indefinite pronouns.’ For instance, Chierchia (2013) argues that expressions, such as *someone, something* and *anyone, anything* and their cross‐linguistic counterparts, have a common semantic core; much work assumes that expressions such as *no one, nothing* are underlyingly negation merged with an indefinite expression, such as *someone, something* (cf. Jacobs, 1980 and much subsequent work). Furthermore, the expressions that Haspelmath subsumes under the term ‘indefinite pronouns’ are often diachronically related across languages, suggesting that their meanings are closely related (Chierchia, 2013; Jäger, 2010; Roberts & Roussou, 2003). While we can thus have some confidence in the reality of the category of indefinite pronouns, the task of justifying the categories posited must be addressed in any work of this kind. This task becomes more pressing in domains of functional vocabulary. In content word domains (e.g., color terms), one can use criteria about the kinds of entities referred to by the expression (e.g., colors) to demarcate the subsystem; but functional domains have more abstract meaning spaces, which makes these criteria harder to apply in practice.

### 7.2 Meaning space of indefinite pronouns

Investigating how efficiently systems of indefinite pronouns cover a certain meaning space presupposes that we have a good understanding of what the meaning space consists of.

We have described in Section 2 the six semantic flavors assumed to constitute the meaning space of indefinite pronouns. As indicated there, however, in doing so, we have abstracted away from a number of subtle differences in syntactic distribution—especially in the domain of items with negative polarity and negative indefinite flavor—which may very well reflect subtle meaning differences. For instance, among negative polarity indefinites, there is a bewildering diversity in syntactic and semantic behavior of various expressions, as evidenced by the literature on strong, weak, and Bagel problem negative polarity items (Krifka, 1994; Pereltsvaig, 2004, a.o.). The situation is similar in the domain of free choice and negative indefinites (Chierchia, 2013; Zeijlstra, 2004). It is possible that future work on semantic correlates of these various types of negative polarity, free choice, and negative indefinites will lead to a more fine‐grained meaning space for indefinite pronouns.

### 7.3 Measures of complexity and communicative cost of languages

The results reported in this paper are robust across four settings: {literal cost, pragmatic cost} × {2‐operators complexity, 3‐operators complexity}, as evidenced by the results of additional experiments reported in Section 6. Other settings may, however, be explored in future work, which may prove to be a more accurate way of measuring communicative cost and complexity.

For instance, to construct a complexity measure for the systems of indefinite pronouns, we have built on the theory of features of indefinite pronouns put forward in Haspelmath, 1997. To our knowledge, Haspelmath's is the only feature‐based theory intended to account for all of the semantic flavors of indefinite pronouns discussed here. There are, however, more recent approaches to the feature content of some of the subcategories of indefinite pronouns, such as negative polarity and free choice indefinites (see, for instance, Chierchia, 2013). These recent proposals may be developed further to construct alternative measures of complexity. Furthermore, while we explored two choices of set operations for features ((i) union and intersection; (ii) union, intersection, and set difference), other choices are conceivable — we do not have a definite answer to the question of which such choice is the cognitively realized one. Finally, one can conceive of measures of complexity not based on features, but, for instance, solely on the number of items in the system.

Similarly, one may consider alternative measures of informativeness. In this respect, it is important to point out a complication in the present approach to measuring communicative cost. The measure of communicative cost incorporates the need to communicate different flavors; in the present, and much other work (e.g., Kemp and Regier (2012)), the communicative need for different flavors is inferred from the frequency of use of different items, which denote those flavors in corpora. However, the frequency of use of different items may depend not only on the communicative need of the flavors they can express, but also on the items’ complexity. If this is indeed the case, there is a bias in the trade‐off analyses such as the one developed in Kemp and Regier (2012) and much related work, including the present one: more complex items in languages would tend to denote flavors which are less commonly expressed, contributing to the overall better simplicity/informativeness trade‐off in such languages (cf. a related discussion in Enguehard and Spector (2021)). At present, it is not clear how to circumvent this issue.

## 8 Conclusions

We find that natural languages optimize the simplicity/informativeness trade‐off in how they organize their indefinite pronoun systems. These results represent an extension of efficiency analyses to a system of function words, thus tying in with Steinert‐Threlkeld, 2019, 2021; Mollica et al., 2021; Zaslavsky et al., 2021; Uegaki, 2022 in concluding that similar communication pressures are shaping both content and function word categories across languages.

Furthermore, we find that there is quite some variability in terms of what the closest point on the Pareto frontier would be for natural languages' indefinite pronoun systems in Experiment 1. Going back to the question of what causes variation between natural languages, this result suggests that some of the variation is due to languages finding different solutions to the problem of simplicity/informativeness trade‐off optimization. Finally, in Experiment 2, we find that artificial languages satisfying Haspelmath's universals are better at trading simplicity and informativeness than languages which do not. In other words, there is a correlation between languages' satisfying Haspelmath's universals and their optimizing of the simplicity/informativeness trade‐off. This suggests that the simplicity/informativeness trade‐off optimization may explain some of Haspelmath's universals. A question that remains for future work is to find out which of Haspelmath's universals can be explained with the trade‐off optimization: it is conceivable that only a proper subset of them can, and that the rest need a different explanation.

Finally, we have discussed the assumptions required at each step of this efficiency analysis in the case of indefinite pronouns. These considerations must be made in any such analysis, and a thorough discussion of the choice points can help illuminate future work in this and related domains.

## Conflicts of interest

The authors have no conflicts of interest to disclose.

## Additional information

An earlier and reduced version of the present work was published as Denić, Steinert–Threlkeld, and Szymanik (2021).

## Notes

1 We note that the term *indefinite pronoun* is not a standard term for these expressions in the formal semantics literature. However, as Haspelmath's (1997) work on these expressions is well known and widely cited, we have opted for keeping the term. Roughly, in more standard semantic terminology, the expressions categorized as indefinite pronouns by Haspelmath (1997) include “vanilla” existential indefinites, negative polarity indefinites, epistemic indefinites, free choice indefinites, and negative indefinites (including negative concord indefinites and negative quantifiers).

2 These data are available in an online Appendix to the paper at https://github.com/milicaden/indefinite-pronouns-simplicity-informativeness.

3 The features $SE^{+}$ and $R^{+}$ are considered to characterize the negative indefinite flavor for two reasons. First, Haspelmath considers these two features to characterize the direct negation function, and as discussed, all indefinite pronouns with the negative indefinite flavor are indexed by Haspelmath as having the direct negation function. Second, these two features characterize the negative polarity flavor, and at least certain negative indefinites (the so‐called negative concord items or N‐words) have been argued to simply be a special type of negative polarity indefinites (i.e., negative polarity items which can induce the presence of negation); see, for instance, Chierchia, 2013.

4 We consider pragmatic speakers and listeners later in the paper.

5 The scripts and data files used for the experiments reported in this paper can be found at https://github.com/milicaden/indefinite-pronouns-simplicity-informativeness.

6 Comparable experiments may be conducted for other ontological categories, such as thing, time, place, and so on. What may change in these additional experiments are the prior probability distributions over flavors and the inventory of indefinite pronouns for natural languages, as natural languages may have gaps in their indefinite pronoun paradigms for certain ontological categories.

7 Recall that all items which convey the negative indefinite flavor, such as English *no one*, are indexed as having *direct negation function* by Haspelmath (but they are not the only items to be indexed as having *direct negation function*). As we relied on acceptability in negative fragment answers to be the distinguishing test for the negative indefinite flavor, we consider the *negative fragment answer* to be the 10th function, and add an extra universal to Haspelmath's list: if an indefinite pronoun can have the *negative fragment answer* function, it can also have the *direct negation function*.

8 The direction of the matching is not expected to affect our conclusions; indeed, the results of Experiment 2 remain qualitatively the same if *Haspel‐ok* languages are matched to *Not Haspel‐ok* languages.

9 One may expect that the measure of complexity will not differ between the two options (i.e., (i) union and intersection; (ii) union, intersection, and set difference) because of the existence of both positive and negative features; if the negative features are the complements of the positive features, the following equivalences hold, with formulae on both sides of = having the same complexity:

(i) Feature1${}^{+}\setminus$ Feature2${}^{+}$ = Feature1${}^{+}$ ∩ Feature2${}^{-}$
(ii) Feature1${}^{+}\setminus$ (Feature2${}^{+}\cap$ Feature3${}^{+}$) = Feature1${}^{+}\cap$ (Feature2${}^{-}\cup$ Feature3${}^{-}$)
(iii) Feature1${}^{+}\setminus$ (Feature2${}^{+}\cup$ Feature3${}^{+}$) = Feature1${}^{+}\cap$ (Feature2${}^{-}\cap$ Feature3${}^{-}$)

However, $R^{-}$ is not the complement of $R^{+}$, and furthermore, the R feature (both + and −) always goes together with the $SE^{+}$ feature (this assumption is inherent to Haspelmath's feature theory, cf. Section 3.1), which means that these equivalences would not hold when the $R^{+}$ or $R^{-}$ feature is involved. Consequently, language complexities may differ between the two options (i.e., (1) union and intersection; (2) union, intersection, and set difference).

10 In the basic RSA model, unlike in (16), the complexity of an expression plays a role in the pragmatic speaker's ($S_{1}$'s) utility. We depart from this in order to be able to evaluate informativeness of the language independently of the language's complexity. In other words, the communicative scenario being modeled is that of interlocutors whose language production and comprehension are not influenced by the complexity of different expressions.
